# *MicroRNA-326* as a Tumor Suppressor Regulates the *NOB1* Expression in Breast Cancer

**DOI:** 10.1155/ijbc/4786830

**Published:** 2026-07-22

**Authors:** Milad Noei, Flora Forouzesh, Fereshteh Abbasvandi, Amirhossein Zare

**Affiliations:** ^1^ Department of Genetics, TeMS.C, Islamic Azad University, Tehran, Iran, azad.ac.ir; ^2^ ATMP Department, Breast Cancer Research Center, Motamed Cancer Institute, ACECR, Tehran, Iran, acecr.ac.ir

**Keywords:** breast cancer, *miR-326*, *NOB1*

## Abstract

**Background:**

Aberrant expression of *miR-326*, a microRNA involved in tumor suppression, has been associated with a broad range of diseases, including cancer and autoimmune disorders. The *NOB1* gene (*NIN One Binding Protein 1 Homolog*) is a critical component that plays a pivotal role in the biogenesis and proper functioning of the 26S proteasome. Researchers have found that *miR-326* acts as a regulator of *NOB1* expression by targeting its mRNA, ultimately leading to a decrease in *NOB1* protein translation.

**Methods:**

This study seeks to assess the expression levels of *miR-326* and *NOB1* genes within breast cancer (BC) tumor tissue, along with an examination of their potential correlation, in comparison to both normal‐adjacent (NATs) and healthy tissues. Forty‐one BC, NATs, and eight healthy breast tissues were used. To compare the expression profiles of *miR-326* and *NOB1* in different tissue groups, including BC, NATs, and healthy tissues, quantitative real‐time PCR (qRT‐PCR) was used.

**Results:**

*miR-326* expression was significantly decreased in BC tissues compared with NATs (*p* < 0.0001) and healthy tissues (*p* < 0.0001). The expression levels of *NOB1* were increased significantly in BC tissue compared with NATs (*p* < 0.0001) and healthy tissues (*p* < 0.0001). A significant negative correlation was observed between *miR-326* and *NOB1* expression (*p* < 0.005). Receiver‐operating characteristic (ROC) curve analysis showed that AUC = 0.8349 and AUC = 0.7 are for *miR-326* and *NOB1*, respectively.

**Conclusion:**

Decreasing the expression of *miR-326* in patients with BC may affect its target gene (*NOB1*) and probably lead to an increase in the expression of the *NOB1* gene. This negative correlation suggests a potential regulatory role for *miR-326* in controlling *NOB1* expression. Our findings demonstrate that dysregulation of the *miR-326* and *NOB1* expression levels may contribute to BC development and progression, indicating the potential of these two genes as biomarkers for diagnostic and therapeutic approaches in BC.

## 1. Introduction

Breast cancer (BC) is the most commonly diagnosed type of cancer in women, with one in eight women worldwide having a lifetime risk of developing the disease. A report estimated that by 2030, 2.3 million people around the globe will be affected by BC. Additionally, among 7.8 million women globally, BC accounted for nearly 12% of all new cancer cases identified in 2020 [1]. For men, BC is responsible for about 0.8%–1% of all cancer cases [2]. Despite significant advancements in treatment, there remains a need for improved diagnostic and prognostic markers [3]. Recent studies have highlighted the importance of inflammation and nutritional status in cancer prognosis, with novel prognostic markers like the Royal Marsden Hospital (RMH) score emerging as promising tools. The RMH score, based on easily accessible clinical and blood parameters such as albumin and LDH levels, has shown a significant association with overall survival across various cancer types and is being increasingly integrated into clinical trials [4]. Breast tumors develop from ductal hyperproliferation and can evolve into benign tumors or metastatic carcinomas [3]. The majority of BCs are readily treatable with surgery, offering a positive outlook. However, a distinct group, roughly one in four, presents a hidden danger. These cancers grow slowly but can spread early, making them particularly challenging [5]. Molecular methods for early detection of BC include circulating tumor (cell‐free) DNA tests and next‐generation sequencing panels. These assays have the potential to facilitate the diagnosis, molecular analysis, and detection of resistance mutations in BC [6]. Recent advancements in targeted therapies and personalized medicine have significantly transformed the treatment paradigm, particularly for subtypes like triple‐negative breast cancer (TNBC), which has been shown to benefit from novel immunotherapeutic approaches. Furthermore, studies have indicated that the integration of targeted medicines with traditional chemotherapy may enhance therapeutic efficacy and improve patient outcomes, which highlights the importance of ongoing research in this area [7]. Furthermore, it has been demonstrated in several studies that new developments in immunotherapy, particularly the use of immune checkpoint inhibitors (ICIs), have a significant influence on the treatment of various types of cancer, such as several aggressive subtypes of BC, including TNBC, a cancer subtype traditionally associated with poor prognosis. Studies have highlighted that TNBC is more likely to respond to ICIs due to the presence of tumor‐infiltrating lymphocytes (TILs) and higher expression of programmed death‐ligand 1 (PD‐L1) in these tumors. These findings suggest that combining ICIs with chemotherapy could improve treatment outcomes for patients with TNBC. MicroRNAs (miRNAs) are small, noncoding RNA molecules that have a significant impact on gene expression regulation. They have a profound impact on various cellular functions, including cell differentiation, proliferation, and apoptosis. Improper miRNA regulation has been associated with a diverse array of diseases, including cancer. Understanding the functions and mechanisms of miRNAs is an active area of research with potential applications in the development of novel therapeutics for various diseases [8]. One of the main roles that miRNAs play in the posttranscriptional processes of regulating gene expression is the degradation of templates and translational repression that result from their complementary interactions with their locations in the 3 ^′^UTRs of target genes [9]. *miR-326*, a small noncoding RNA molecule, has been implicated in a range of biological processes, including neuronal differentiation, immune system modulation, and tumor suppression [10]. Interestingly, *miR-326* suppresses cell growth and invasion in human liver cancer by activating the apoptosis signaling pathway. Studies have shown that *miR-326* expression levels are dysregulated in different types of cancer, such as glioma, melanoma, and hepatocellular carcinoma (HCC), and that restoring its expression can inhibit tumor cell proliferation and invasion [10]. Furthermore, this miRNA is involved in various cellular processes such as tumor metastasis and tumor proliferation by targeting specific genes like *Adam17, NSBP1, Phox2a, FSCN1, CCND1, NOB1* (*NIN1/RPN12* binding protein 1 homolog), and *B7-H3* in a diverse range of diseases, including NSCLC (non‐small cell lung cancer) and BC. Moreover, *miR-326* has a pivotal role in signaling pathways, such as *Hh/Gli, PI3K/Akt*, and *RAS/ERK*, and drug resistance activities by regulating the expression of the *ABCC1* gene in HCC [10]. Additionally, *miR-326* has been investigated as a potential therapeutic target for autoimmune diseases and neurodegenerative disorders such as multiple sclerosis and Alzheimer′s disease [11, 12]. Ji et al. found that *miR-326* has a role in different biological processes within cells, thus making it a potential target for therapeutic purposes in cancer and various other diseases by inhibiting cell growth and migration through targeting specific genes such as *NOB1*. They realized that the expression level of *miR-326* is significantly reduced in patients with gastric cancer (GC) as well as in the cell lines MKN‐28, NCI‐N87, MKN‐45, and AGS when compared with control groups [13].

*NOB1* participates in the control of various cellular processes. It was initially identified as a subunit of the regulatory particle of the 26S proteasome, which is responsible for the degradation of ubiquitinated proteins. Moreover, *NOB1* contributes to the regulation of RNA metabolism, specifically in the processing and maturation of ribosomal RNA (rRNA). In recent years, *NOB1* has been associated with the development and progression of different cancer types [14]. This gene, that is found in eukaryotes and archaea, is essential for the final maturation of small subunit rRNA in yeast, where it facilitates cleavage at site D following the export of the preribosomal subunit into the cytoplasm [15]. It has also been observed that this gene serves an oncogenic function in ovarian cancer and acts as a marker gene for identifying chronic myeloid leukemia, suggesting that it may contribute to the invasion and metastasis of tumor cells. In addition, *NOB1* uses its endonuclease activity to catalyze the cleavage of RNA substrates. It also plays a role in ribosome synthesis and is linked to various cellular processes associated with cancer [16–18]. The upregulation of *NOB1* expression has been observed in various cancers, such as BC, lung cancer, and HCC [19–21]. In BC, increased *NOB1* expression has been linked to a negative prognosis and heightened metastatic potential [19]. Similarly, in patients with *NSCLC*, high levels of *NOB1* expression have been associated with tumor development [22]. A study demonstrated that knockdown of *NOB1* increased apoptosis and reduced proliferation in *NSCLC* cell lines, indicating that *NOB1* plays a crucial role in promoting tumor growth and survival [23]. Furthermore, a positive correlation was observed between *NOB1* expression and tumor size, vascular invasion, and overall poor survival [24–26]. In cell line studies, the inhibition of *NOB1* expression was found to inhibit the proliferation and migration of *HCC* cells, suggesting that *NOB1* plays a critical role in the development and progression of *HCC* [19, 21, 27]. Nevertheless, *NOB1* has been observed to interact with several vital signaling pathways involved in cancer development and progression. For instance, NOB1 has been found to interact with the JNK signaling pathway, which is known for regulating cell proliferation, migration, and cell cycle arrest, and is closely related to the cell survival and metastasis of cancer cells. Activation of the JNK signaling pathway has context‐dependent functions, as it can regulate apoptosis through phosphorylation of proteins involved in survival signaling. Accordingly, it is suggested that dysregulation of NOB1 expression may contribute to cancer development, at least in part, through alteration of JNK‐mediated signaling pathways [28]. To determine the protein interactions in which *NOB1* engages, the protein–protein interactions (PPIs) method can be used. This technique represents complex biological functions that occur when two or more proteins physically interact with one another. Several diseases and some potential drug targets have been identified using PPIs by scientists. To gain a deeper understanding of the functions of proteins that have not yet been discovered, computational analysis of PPI networks is becoming increasingly important. As a result of the development and progress of modern systems biology, PPI has become one of the key topics to consider [29].

The primary objective of this study was to explore the correlation between *NOB1* and *miR-326* expression levels in BC patients. Previous studies have demonstrated the dysregulation of *miR-326* and *NOB1* in various cancers, suggesting their involvement in tumorigenesis and a direct regulatory relationship. We compared the expression levels of these two molecules in tumor tissues, normal adjacent tissues (NATs), and healthy tissues. Our findings indicated that *miR-326* was downregulated and *NOB1* mRNA was upregulated in BC patients.

## 2. Materials and Methods

### 2.1. Patients and Tissue Samples

The Iran National Tumor Bank at Imam Khomeini Hospital provided fresh tissue samples from 41 women with BC. Each sample contained both tumor and normal‐adjacent tissue, obtained from within about 3 cm of the tumor. These samples were stored in a freezer at −70°C until RNA extraction was performed. Normal breast tissues of eight healthy women were obtained undergoing cosmetic surgery and served as the control group. These tissues were immediately placed on ice, and immediately after being sent to the laboratory, RNA extraction was performed. The average age of the BC patients was approximately 48 years (48.61 ± 12.13) (aged 29–81 years). Patients did not receive any chemotherapy or radiotherapy. To verify the validity of the study findings, the pathologist independently reviewed and confirmed the diagnosis, histological grade, and clinical stage for each case. This study was approved by the Ethics Committee of Islamic Azad Tehran Medical Sciences University, Pharmacy and Pharmaceutical Branches Faculty, Tehran, Iran (Ethics Approval Number: https://ir.iau.ps.rec.1397.170/). Written informed consent was obtained from all participants prior to tissue collection.

### 2.2. Extraction of RNA and complementary DNA (cDNA) Synthesis

According to the manufacturer′s instructions, total RNA was extracted from all types of fresh tissues, including tumor tissue, NAT, and healthy tissue, using the NucleoSpin RNA purification kit (MACHEREY‐NAGEL, Germany). Approximately 30 mg of tissue was homogenized in 350 *μ*L of RA1 buffer supplemented with 3.5 *μ*L of *β*‐mercaptoethanol to inactivate RNases. The homogenate was centrifuged to remove cell debris, and the supernatant was transferred to a collection tube containing a filter. After centrifugation, the flow‐through containing the RNA was transferred to a new tube and mixed with 70% ethanol. The RNA was then bound to a silica membrane within a spin column. To remove contaminating genomic DNA, on‐column DNase I digestion was performed. Following DNase inactivation, the column was washed with buffers RA2 and RA3 to remove salts, proteins, and other contaminants. Finally, the purified RNA was eluted in RNase‐free water. The concentration and purity of the isolated RNA were evaluated using a NanoDrop spectrophotometer by measuring absorbance at 260 and 280 nm, and ratios between 1.8 and 2 were considered acceptable.

Following the removal of DNA contamination with DNase‐1, the PrimeScript RT Reagent Kit (Takara, Japan) was used to synthesize cDNA strands under the manufacturer′s instructions. cDNA was synthesized with a stem‐loop primer to measure the expression levels of *miR-326*. DNA concentration was measured using a NanoDrop 2000c spectrophotometer. (Thermo Scientific, Wilmington, Delaware, United States).

### 2.3. Target Genes Prediction of miR‐326

Using the algorithms of miRTarBase https://www.mirtarbase.cuhk.edu.cn/ and TargetScan 7.1 (https://www.targetscan.com/), targets of *miR-326* were predicted and identified.

### 2.4. Quantitative Real‐Time Polymerase Chain Reaction (qRT‐PCR)

The ExiCylcer 96 (Bioneer, Korea) instrument and WizPure qPCR Master (SYBR) (Korea) were used to perform quantitative RT‐PCR. The relative expression of the *NOB1* gene and *miR-326* was normalized to *GAPDH* and U6 expression, respectively. The following specific primers were used: *U6*–F: 5 ^′^–GCTTCGGCAGCACATATAC–3 ^′^, *U6*–R: 5 ^′^–ATTCCGTTTCTGGGAGGG–3 ^′^, *NOB1*–F: 5 ^′^–ATCTGCCCTACAAGCCTAAAC–3 ^′^, *NOB1*–R: 5 ^′^–CGTCCTCACCTCTGTCAATCA–3 ^′^, *GAPDH*–F: 5 ^′^–ATTTGGTCGTATTGGGCG–3 ^′^, *GAPDH*–R: 5 ^′^–GTACTCAGCGCCAGCATC–3 ^′^, *miR-326*–F: 5 ^′^–CCTCTGGGCCCTTCCTCCAGGTCGT–3 ^′^, *miR-UniR*: 5 ^′^–CCAGTGCAGGGTCCGAGGTA–3 ^′^, SL primer: 5 ^′^–GTCGTATCCAGTGCAGGGTCCGAGGTATTCGCACTGGATACGACCTGGAG–3 ^′^. qRT‐PCR was used for the *NOB1* gene and *miR-326* using a 20‐*μ*L reaction mixture: 1‐*μ*L cDNA, 0.5 *μ*L of each 10 pmol/*μ*L primer (Sinaclone, Iran), 10 *μ*L of qRT‐PCR Master (SYBR) (10X), and 8‐*μ*L dH2O, and followed by initial denaturation at 95°C for 5 min, 40 cycles of denaturation at 95°C for 30 s, annealing and extension at 60°C for 30 s, and melting curve analysis in the range of 70°C–95°C for 2 s. To compare the expression levels of different genes, the 2^−*ΔΔ*Ct^ quantitative method was employed, with each sample analyzed twice.

### 2.5. PPI

We used the bioinformatics tool STRING software Version 12.0 (https://www.string-db.org/) in this study to examine the PPI of the *NOB1* gene. STRING is a well‐established database that is useful for identifying known and predicted protein interactions of the *NOB1* gene. This network was used as a bioinformatics, hypothesis‐generating tool to explore potential functional partners of *NOB1*. Experimental validation of these interactions, including Co‐IP assays, was beyond the scope of the current study and warrants future investigation.

### 2.6. Statistical Analysis

The data from the experiments are depicted as the mean ± standard error (SEM). Differences between BC tissues and NATs were analyzed by means of unpaired *t*‐tests. For assessing multiple group comparisons, the study used one‐way analysis of variance (ANOVA) and Tukey′s test. To analyze the correlation between *NOB1* gene expression and *miR-326* levels, Pearson′s correlation method was applied. We considered a *p* value lower than 0.05 to be statistically significant. These statistical analyses were conducted with GraphPad Prism software (GraphPad Software, La Jolla, California, United States).

## 3. Results

### 3.1. miR‐326 Expression Is Downregulated in BC Tissues

**Figure 1:**
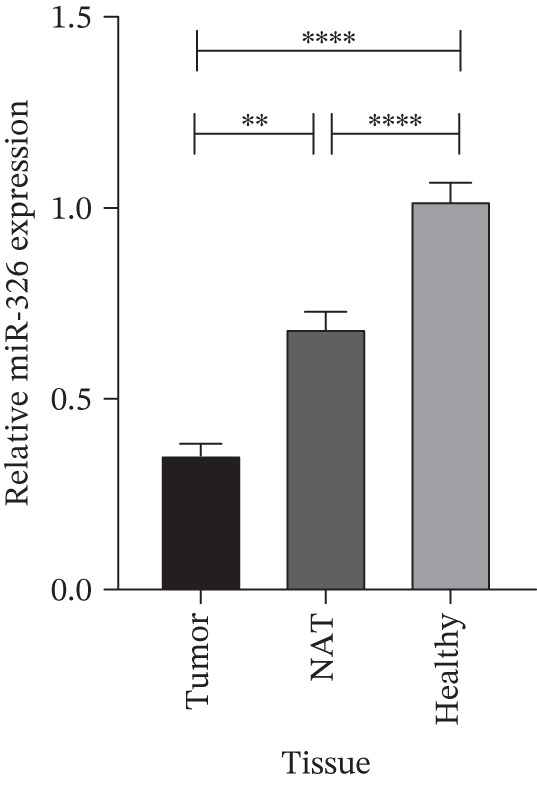
*miR-326* is downregulated in BC tissues and NATs. Relative expression of *miR-326* in BC tissues, NATs, and healthy tissues was determined by qRT‐PCR. The expression level of *micR-326* is significantly downregulated in BC tissues compared with NATs and healthy tissues ( ^∗∗^ *p* < 0.01 and  ^∗∗∗∗^ *p* < 0.0001, respectively). *miR-326* is also significantly downregulated in NATs compared with healthy tissues ( ^∗∗∗∗^ *p* < 0.0001) (means ± SEM: tumor vs. NAT: −0/3283 ± 0/05456, tumor vs. healthy: −0/6600 ± 0/09549, NAT vs. healthy: −0/3317 ± 0/09549). *U6 snRNA* was used as an internal control. Data are expressed as means ± SEM. BC: breast cancer, NAT: normal adjacent tissue, snRNA: small nuclear ribonucleic acid, miR: microRNA, SEM: standard deviation.

qRT‐PCR was employed to measure the expression levels of *miR-326* in both BC and NATs as well as in healthy tissue samples. The expression of *miR-326* in BC tissues was found to be significantly lower when compared with its expression in healthy tissues. (*p* < 0.0001) and NATs (*p* < 0.001). The expression levels of *miR-326* in NATs showed a significant downregulation compared with healthy tissues (*p* < 0.005) (Figure 1). To gain further insights, we examined the potential association between *miR-326* expression and key clinical data, including tumor grade, cancer stage, and patient age (check Table 1). Our findings suggest that the expression level of *miR-326* is not significantly associated with clinicopathological characteristics such as patients′ age, histological grades, and clinical stages.

**Table 1:** Clinicopathologic characteristics of patients with BC.

Characteristics	Number of patients	Number analyzed (%)
Age at disease onset (year)		
≥ 50	27	65.85
< 50	14	34.14
Clinical stage		
Stage *Ι*	3	7.31
Stage *ΙΙ*	26	63.41
Stage *ΙΙΙ*	12	29.26
Histological grade		
Grade *Ι*	3	7.31
Grade *ΙΙ*	27	65.85
Grade *ΙΙΙ*	11	26.82

### 3.2. NOB1 Is a Target Gene of miR‐326

Two widely used software programs, TargetScan and miRTarBase, helped us delve into the potential mechanisms of *miR-326* (Accession: MIMAT0000756). We identified *NOB1* as a possible target because it possesses a binding site in its 3 ^′^‐untranslated region (UTR) that could potentially interact with *miR-326*.

**Figure 2:**
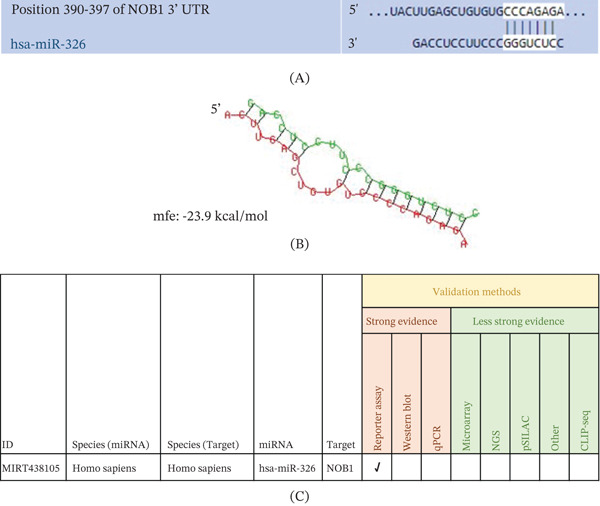
*NOB1* is a direct target of *miR-326* in breast cancer. (A) *NOB1* 3 ^′^‐UTR contains the target sites where *miR-326* binds. (B) *miR-326* putatively targets the 3 ^′^‐UTR of *NOB1* mRNA. Green strand: *miR-326*, red strand: 3 ^′^‐UTR of *NOB1* mRNA. (C) *NOB1* was confirmed as a target of *miR-326* by miRTarBase.

Through bioinformatics prediction, potential binding sites of *miR-326* on *NOB1* mRNA were identified. *NOB1* was determined to be the target gene for *miR-326* using TargetScan software (https://www.targetscan.com/) (Figure 2)A and miRTarBase (https://www.mirtarbase.cuhk.edu.cn/ (Figure 2)B due to the presence of a putative *miR-326* target site in its 3 ^′^‐UTR. It was confirmed by using RNAhybrid 2.2 software (https://bibiserv.cebitec.uni-bielefeld.de/rnahybrid) that *miR-326* is predicted to targetthe 3 ^′^‐UTR of *NOB1* with a relatively strong interaction. It indicates a stronger connection between miRNA and its target gene if more base pairs in the 5 ^′^ region of miRNA are bound together by a hydrogen bond (Figure 2)C. By using IntaRNA Version 3.3.1 (https://rna.informatik.uni-freiburg.de/IntaRNA), the interaction between *miR-326* and the 3 ^′^ UTR region of *NOB1* mRNA was predicted as well. For *miR-326* and *NOB1* interactions, a minimal energy profile is given. The minimum energy of any possible RNA‐RNA interaction that can form between these interactions is −9.46 kcal/mol, the hybridization energy is −16.44 kcal/mol, and the unfolding energy of *NOB1* mRNA and *miR-326* is 3.33 kcal/mol and 3.65 cal/mol, respectively.

### 3.3. NOB1 mRNA Expression Is Upregulated in BC Tissues

**Figure 3:**
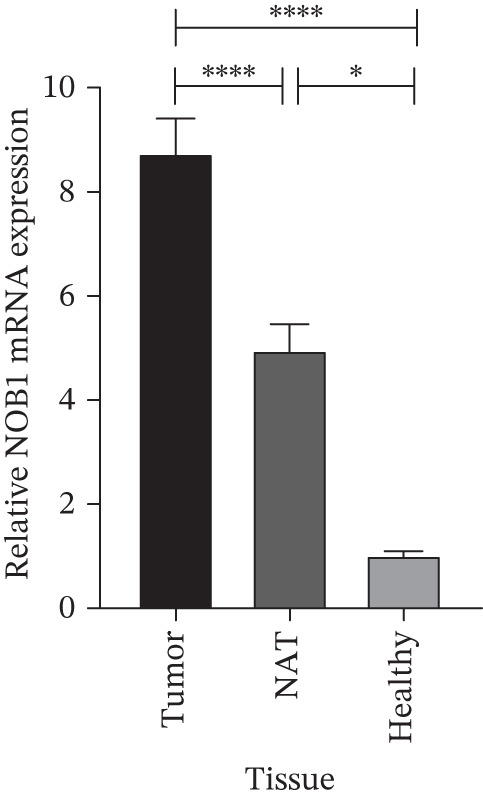
*NOB1* mRNA expression is upregulated in BC tissues and NATs. Relative expression levels of *NOB1* mRNA were examined by quantitative RT‐PCR in BC tissues, NATs, and healthy tissues. The expression of *NOB1* mRNA is upregulated in BC tissues and NATs compared with healthy tissues ( ^∗∗∗∗^ *p* < 0.0001). There is an upregulation of the expression level of *NOB1* in NATs compared with healthy tissues ( ^∗^ *p* < 0.05) (means ± SEM: tumor vs. NAT: 3/757 ± 0/8586, tumor vs. healthy: 7/663 ± 1/503, NAT vs. healthy: 3/906 ± 1/503). To normalize expression levels, *GAPDH* was utilized as an internal control. Data were reported as mean ± SEM. BC: breast cancer, NAT: normal adjacent tissue, *GAPDH*: *glyceraldehyde-3-phosphate dehydrogenase*, *NOB1*: *NIN One Binding Protein 1 Homolog*, mRNA: messenger ribonucleic acid, SEM: standard deviation.

We used quantitative *RT-PCR* to investigate the expression levels of *NOB1* mRNA in BC tissues, NATs, and healthy tissues. We observed that the expression level of *NOB1* mRNA was significantly upregulated in BC tissues and NATs compared with healthy tissues as the control group (*p* < 0.0001 and *p* < 0.0001, respectively). This gene′s level of expression is also markedly upregulated in NATs compared with healthy tissues (*p* < 0.05) (Figure 3). Next, we examined whether *NOB1* expression aligned with any variations in the patients′ clinical characteristics, including histological grade, clinical stage, and age. Our observations indicate that the expression of *NOB1* mRNA is not significantly related to clinicopathological features such as histological grade, clinical stages, and patients′ age.

### 3.4. PPI

**Figure 4:**
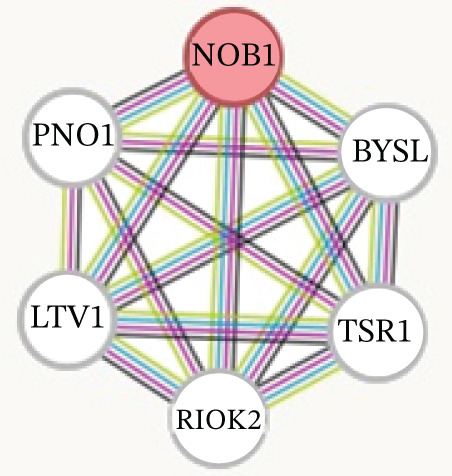
Proteins are represented as network nodes using the STRING bioinformatics tool. Posttranslational modifications and splice isoforms are collapsed, meaning that every node reflects all the proteins generated by a single protein‐coding gene locus. Black lines connecting two circles represent the coexpression relationship between the two proteins; purple and blue lines are those whose interactions are experimentally determined and extracted from curated databases, respectively; and yellow lines connecting two proteins indicate the relationship obtained through textmining. The red node is the query protein and the first shell of interaction, and the white nodes are the second shell of interactions.

The possible network interactions of the *NOB1* protein in *Homo sapiens*, including both functional and physical protein associations, are illustrated in Figure 4. The *NOB1* protein, as the query one, is demonstrated by its red color. This network is based on the highest confidence interaction score of 0.900, and each node represents a protein; the connections between nodes (lines) can be seen in different colors, indicating the type of interaction evidence. Various sources were used to determine the interaction of the *NOB1* protein, including textmining, other experiments, databases, coexpression, neighborhood, gene fusion, and co‐occurrence. By revealing new pathways for additional experimental evaluations, the STRING network helps us understand the known and expected connections of the *NOB1* protein within biological processes.

Each interaction record of the *NOB1* protein with the top ten predicted proteins has been given a score (< 1). These records are imported from several primary interaction databases, lab experiments, and text mining. For example, protein BYSL is ranked first with a score of 0.999, and protein RPS17 is ranked 10th with a score of 0.994.

### 3.5. Negatively Correlation Between the Expression of miR‐326 and NOB1

**Figure 5:**
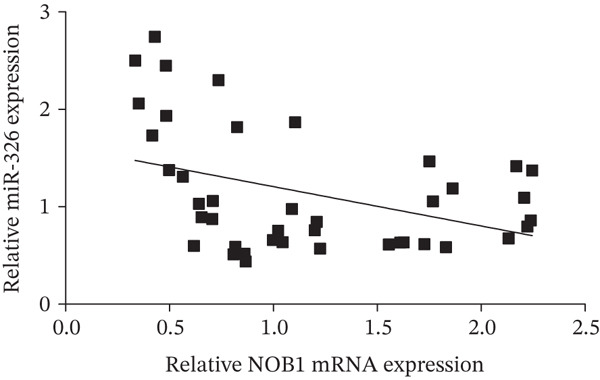
*miR-326* significantly regulates the expression of *NOB1* by binding to the *NOB1* 3 ^′^‐UTR in breast cancer tissues. (Slope =  ^′^0.4039, *p* = 0.0099). The Pearson correlation method was used to determine the relationship between *miR-326* and *NOB1* expression levels.

To investigate the relationship between *miR-326* and *NOB1* expression in BC tissues, we conducted a correlation analysis. Our findings revealed a significant negative correlation between *miR-326* and *NOB1* expression in BC tissues (slope = −0.4039, *p* = 0.0099). Accordingly, the expression of *NOB1* levels is increased when *miR-326* is downregulated. These data support the notion that *miR-326* potentially targets *NOB1* in BC tissues by binding to its 3 ^′^‐UTR, ultimately leading to the mRNA of *NOB1* degradation or translational inhibition (Figure 5).

### 3.6. Diagnostic Significance of miR‐326 and NOB1 Expression Profiles

**Figure 6:**
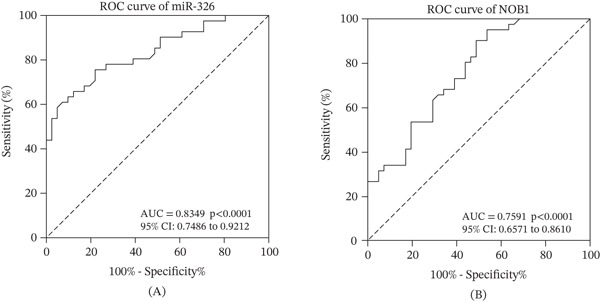
Evaluating the potential of *miR-326* and *NOB1* as diagnostic biomarkers to distinguish between BC patients and healthy tissues using ROC curve analysis. (A) The expression level of *miR-326* was effective in discriminating BC patients from healthy individuals (AUC = 0.8349, *p* < 0.0001). (B) *NOB1* expression level was a significant biomarker for differentiating BC patients from healthy individuals (AUC = 0.7591, *p* < 0.0001).

We investigated the ability of specific miRNAs to discriminate between BC and NATs using ROC analysis. Higher AUC values indicate a more reliable test for differentiating these two tissue types. Notably, *miR-326* (AUC = 0.8349, *p* < 0.0001) and *NOB1* (AUC = 0.7591, *p* < 0.0001) emerged as promising candidates for distinguishing BC patients from healthy individuals, suggesting their potential as biomarkers (healthy tissues and NATs) (Figure 6A, Figure 6B, respectively). In order to determine the optimal cutoff point, we used the Youden index. Based on this index, our optimal and desirable cut‐off point is the one with the highest sum of sensitivity and specificity. Our findings revealed that the sensitivity and specificity of *miR-326* as a biomarker were 75.61% and 78.05%, respectively. Additionally, the sensitivity and specificity of *NOB1* as a biomarker were 90.24% and 51.22%, respectively. The AUC, 95% confidence interval calculated by the Wilson/Brown method. A 95% CI ratio of *miR-326* was 0.7486–0.9212, and 95% CI ratio of *NOB1* was 0.6571–0.8610.A.

## 4. Discussion

Studies have demonstrated the diverse roles of *miR-326*, exploring its expression levels in various diseases. This miRNA exhibits significant roles in fundamental cellular processes, acting as a regulator of cell proliferation, invasion, apoptosis, signaling pathways, and even tumorigenesis. Its regulatory role is due to its ability to bind specific messenger RNAs at their 3 ^′^ UTR, either suppressing their translation or triggering their degradation [10]. *miR-326* regulates a variety of biological processes within cells, making it a potential therapeutic target in cancer and other diseases. Ji et al. discovered that the expression level of *miR-326* is downregulated in patients with GC and *MKN-28*, *NCI-N87*, *MKN-45*, and *AGS* cell lines in comparison to control groups. This research has implicated *miR-326* as a potential tumor suppressor, inhibiting cell migration, invasion, and growth through specific gene targets like *NOB1* [13]. Our study adds to this evidence, suggesting that decreased expression levels of *miR-326* may have a role in tumorigenesis and progression in BC by regulating *NOB1* expression. Notably, we found a decrease in *miR-326* levels in breast tumor tissues compared with NATs and healthy tissues, suggesting that its downregulation might be associated with tumor invasion and its advancement. Our results showed that the changes in molecular mechanisms occur from NATs and the expression of genes was different from healthy tissues. NATs are influenced by the tumor microenvironment (which healthy tissues lack), characterized by growth factors, cytokines, and other signaling molecules that can change gene expression. Although NATs are histologically nonmalignant, multiple transcriptomic studies have shown that they occupy an intermediate molecular state between healthy and tumor tissues, exhibiting significant gene expression alterations that are neither fully normal nor fully tumor‐like. Such changes are thought to be driven by proinflammatory signals, stromal remodeling, and other influences from the tumor microenvironment, consistent with the concept of “field cancerization” [30]. Accordingly, the altered expression patterns observed in NATs, including downregulation of *miR-326* and upregulation of *NOB1*, may reflect early molecular changes influenced by the tumor microenvironment rather than classical malignant transformation. Compared with healthy tissues, NATs have early genetic alterations that dispose them to malignant transformation. The present study was considered to compare the expression of *miR-326* and *NOB1* between tumor tissues and NATs and compare them with healthy tissues to better understand the changes of these genes in BC and their biomarker potential, but more studies are needed. Yu et al. showed that *miR-326* was downregulated as a target of Hsa_circ_0003998 in patients with lung adenocarcinoma and *A549/DTX* and *H1299/DTX9* cell lines compared with control groups [31]. In 2016, Cao et al. found out that *miR-326* is significantly downregulated in patients with osteosarcoma (solid tumor and serum) who received neither chemotherapy nor radiotherapy compared with NATs as the control group. The same result was observed in *HOS*, *U2OS*, and *MG-63* cell lines in comparison with the hFOB (human fetal osteoblastic) cell line as the control group [32].

Wang et al. showed that the downregulation of *miR-326* is associated with a poor prognosis and reduced overall survival in lung cancer patients. The overexpression of *miR-326* in lung cancer cells decreased cellular proliferation, migration, and invasion by inhibiting oncogenic targets such as *phox2a* [33]. Examining the potential interaction between *miR-326* expression and various clinical parameters, including histological grade, clinical stage, and age, our study did not find any statistically significant associations. This miRNA can regulate angiogenesis, invasion, and migration of cancer cells by targeting genes involved in these processes. Our bioinformatics analysis showed that *NOB1* is one of the target genes of *miR-326*. *NOB1* is involved in the synthesis of ribosomes in both yeast and archaea. Through its catalytic cleavage at site D following the export of the preribosomal subunit into the cytoplasm, it contributes to the ultimate maturation of the small subunit rRNA in yeast [34]. Additionally, *NOB1* utilizes its endonuclease activity to cleave RNA substrates. As a result, it participates in the synthesis of ribosomes and is connected to several cancer‐related cellular processes [16].

We reported that the expression levels of the *NOB1* gene were upregulated significantly in BC tissues and NATs compared with healthy tissues. According to these results, tumor invasion and cancer development may be caused by increased expression levels of the *NOB1* gene in BC tissues as compared with control groups (NATs and healthy tissues), making it a potential prognostic biomarker. *miR-326* regulates the expression levels of *NOB1* by binding to its 3 ^′^‐UTR and inhibiting its transcription or translation [35]. The interaction between *miR-326* and *NOB1* is important for the regulation of ribosome biogenesis, as *NOB1* is a factor in the processing and maturation of rRNA [36]. Li et al. reported that in BC, *NOB1* overexpression has been associated with poor prognosis and increased metastatic potential. *NOB1* expression was found to be significantly higher in metastatic BC compared with nonmetastatic tumors [19]. Similarly, in another study on patients with laryngeal carcinoma done by Gao et al., NOB1 has been shown to interact with several key signaling pathways that are involved in cancer development and progression. For example, NOB1 has been found to interact with the JNK signaling pathway, which is a key mediator of cell proliferation, migration, and apoptosis. NOB1 knockdown was found to activate the JNK signaling pathway, suggesting that NOB1 plays a key role in regulating this pathway [28]. *NOB1* knockdown increased apoptosis and decreased proliferation in *NSCLC* cell lines, suggesting that *NOB1* plays a key role in promoting tumor growth and survival. Furthermore, *NOB1* expression was found to be associated with tumor size, vascular invasion, and poor overall survival [24–26]. Our study revealed the significant upregulation of the *NOB1* gene in BC tissues compared with control groups (NATs and healthy tissues). Despite examining potential links between *NOB1* levels and various clinicopathological features like tumor grade, cancer stage, and age, our analysis did not identify any statistically significant associations. This research can potentially enhance our understanding of BC biology, especially in the context of miRNA‐mediated gene regulation. The discovery that *NOB1* and *miR-326* are important factors in the advancement of BC creates new opportunities for therapeutic purposes. When traditional treatments fail to control BC, *miR-326* and its downstream mediators may offer a strategy for combating this disease. Furthermore, the possibility of using *miR-326* as a biomarker for early detection is an intriguing opportunity that, of course, requires further research.

To evaluate the ability of *miR-326* and *NOB1* to serve as diagnostic biomarkers for BC, we utilized ROC curve analysis. Our findings offer that both *miR-326* and *NOB1* may be valuable for identifying BC patients in the future. Although *NOB1* exhibited promising diagnostic metrics, including a significant AUC in ROC analysis, its relatively low specificity in our cohort suggests that it may be limited as a single diagnostic biomarker. Such limitations are consistent with the broader cancer biomarker literature, where individual molecular markers often do not achieve ideal specificity or sensitivity independently. Several studies have shown that combinations of biomarkers, including panels of miRNAs or integrated molecular signatures, can significantly improve diagnostic performance beyond what is achievable by single markers alone [37]. These findings underscore the potential value of *miR-326* and *NOB1*, as diagnostic biomarkers for BC and support the promising possibility of using them in early disease detection. The current data suggest that their utility in prognostic assessment may be limited, highlighting the need for further studies to clarify their prognostic relevance and to validate their clinical applicability. Accordingly, future studies should explore combining *NOB1* with *miR-326* and other biomarkers or clinical indices to enhance diagnostic accuracy. Despite these promising results, there are still some unanswered questions. One of the limitation of our study was the relatively small number of healthy breast tissue samples in the control group. Therefore, future studies with larger control cohorts are required.

Furthermore, although multiple bioinformatics tools consistently predicted *NOB1* as a direct target of *miR-326* and a significant inverse correlation was observed in clinical samples, functional experiments are required to confirm the direct binding of *miR-326* to the 3 ^′^‐UTR of *NOB1* in future studies.

Looking ahead, we expect to see that advances in miRNA‐based therapies are likely to appear, with the development of *miR-326* mimics or inhibitors as potential therapeutic agents for BC. Furthermore, the integration of high‐throughput sequencing and bioinformatics approaches will facilitate the identification of additional *miR-326* targets, providing a more comprehensive understanding of its regulatory network. We also anticipate that the use of *miR-326* as a biomarker for BC diagnosis and prognosis will be improved, potentially leading to its incorporation into clinical practice. Overall, this study contributes to the foundation on which future research will build, ultimately aiming to improve outcomes for BC patients through more targeted and personalized therapeutic strategies.

## 5. Conclusion

The results revealed a downregulation of *miR-326* and an upregulation of *NOB1* in BC tissues, with a significant negative correlation between their expression levels. These findings suggest that *miR-326* and *NOB1* could serve as potential molecular biomarkers for BC, though further research is needed to fully understand their role in tumorigenesis.

## Author Contributions

F.F. designed the study and was responsible for supervising the study. F.A., M.N., and A.Z. were responsible for collecting samples. F.A. helped in clinical consultation. M.N. and A.Z. did the molecular experiments and bioinformatics analysis. F.F., M.N., and F.A contributed extensively to the interpretation of the data and the conclusion. M.N. and A.Z. wrote the first draft of the manuscript. All authors performed editing and participated in the finalization of the manuscript.

## Funding

No funding was received for this manuscript.

## Disclosure

All authors approved the final draft.

## Conflicts of Interest

The authors declare no conflicts of interest.

## Data Availability

Additional data that was not included in the presentation can be obtained upon request.
